# Taxonomic integrative and phylogenetic identification of the first recorded *Triatoma rubrofasciata* in Zhangzhou, Fujian Province and Maoming, Guangdong Province, China

**DOI:** 10.1186/s40249-019-0579-8

**Published:** 2019-08-13

**Authors:** Yue Hu, Min-Zhao Gao, Ping Huang, Hong-Li Zhou, Yu-Bin Ma, Min-Yu Zhou, Shao-Yun Cheng, Han-Guo Xie, Zhi-Yue Lv

**Affiliations:** 1 Zhongshan School of Medicine, Sun Yat-sen University, Guangzhou, 510080 China; 2 Key Laboratory of Tropical Disease Control (Sun Yat-sen University), Ministry of Education, Guangzhou, 510080 China; 3 Provincial Engineering Technology Research Center for Biological Vector Control, Guangzhou, 510080 China; 4 Department of Gastroenterology of the Fifth Affiliated Hospital, Sun Yat-sen University, Zhuhai, 519000 China; 5 Fujian Center for Disease Control and Prevention, Fuzhou, 350001 China

**Keywords:** *Triatoma rubrofasciata*, Molecular identification, Phylogenetic study

## Abstract

**Background:**

Most species of Triatominae live exclusively in Latin America. However, one species, *Triatoma rubrofasciata*, has been recorded in the Americas as well as in various port areas in Africa and Asia. An increasing number of *T. rubrofasciata* have been reported in southern China in recent years. However, the origin of this invasive insect vector in China remains unknown, therefore, accurate identification and phylogenetic analysis of the bugs are urgently needed.

**Methods:**

A total of seven triatomine insect specimens were found and collected from Maoming City, Guangdong Province, China (GDMM) and Zhangzhou City, Fujian Province, China (FJZZ), respectively. The obtained insect vector specimens were observed under a dissecting microscope for morphological classification and then the genomic DNA was extracted, and the 16S ribosomal RNA (rRNA), 28S rRNA as well as cytochrome oxidase subunit I (COI) genes of the species were amplified and sequenced. Subsequently, molecular phylogenetic analyses based on multiple alignments of the above genes were conducted in order to identify the species and determine the phylogenetic origin approximation accurately.

**Results:**

The triatomine insects collected from GDMM and FJZZ were identified as *Triatoma rubrofasciata* using morphological and genetic analyses. All of the Chinese *T. rubrofasciata* captured in FJZZ, GDMM and other localities in southern China, together with a Vietnamese and Brazilian strain, formed a new, cohesive clade. *T. rubrofasciata* in GDMM and FJZZ are likely derived from strains found in Vietnam or Brazil.

**Conclusions:**

To the best of our knowledge, this is the first record of the invasive insect *T. rubrofasciata*, which is likely derived from strains native to Vietnam or Brazil, in both Maoming City, Guangdong Province and Zhangzhou City, Fujian Province of China. A comparison of the DNA sequences of the 16 s rRNA, 28 s rRNA and COI genes confirmed the specific identification of *T. rubrofasciata*, and its potential origin in China is based on the phylogenetic analyses undertaken in this study. More targeted interventions and improved entomological surveillance are urgently needed to control the spread of this haematophagous insect in China.

**Electronic supplementary material:**

The online version of this article (10.1186/s40249-019-0579-8) contains supplementary material, which is available to authorized users.

## Multilingual abstracts

Please see Additional file 1 for translations of the abstract into the five official working languages of the United Nations.

## Background

Triatomines, also known as kissing bugs due to their tendency to bite human faces, are insects from the order Hemiptera and family Reduviidae; they are the main vectors responsible for the transmission of American trypanosomiasis (Chagas disease), a chronic, systemic, tropical parasitic disease caused by *Trypanosoma cruzi* that infects an estimated 8 million people worldwide, mostly in Latin America [1–3]. There are a total of 151 triatomine species (149 extant and two extinct) that are currently composed of five tribes: Aberproseniini, Bolboderini, Cavernicolini, Rhodniini and Triatomini. Most Triatomine occur in the New World; only one genus, *Triatoma*, which is included in the Triatomini, is found in both in the Old and New Worlds [3, 4].

*Triatoma rubrofasciata* is characterized by a triangular scutellum and an orange-red margin along the outer edge of the abdomen and the sides of the pronotum [5]; it is a cosmopolitan species from the genus *Triatoma* and is widely distributed across the globe, although it is most commonly found in Asia, Oceania, Africa and Central America [6–8]. This tropicopolitan insect is also widely spread across the southern coastal areas of China including Guangdong, Guangxi, Hainan and Taiwan [6, 9]. Although Chagas disease has not been reported in China, dermatitis and anaphylactic shock caused by triatomine insects have been recorded since the 1980s [10, 11]. Trends in global vector migrations from endemic to non-endemic countries have greatly increased the risk of Chagas disease expansion to global levels [12]; migration of these insect vectors could help spread this disease beyond its previous limits [13], which suggests there is a potential risk of Chagas disease being introduced and transmitted in China, posing a significant threat to public health [14]. Therefore, accurate identification and phylogenetic analysis of *T. rubrofasciata* will trace back its potential origin and carry out scientific and effective prevention, which may helpful for the control of its spread.

The distinctive features of the triatomine are based primarily on morphological characteristics such as the genitalia [15, 16] and appendages [17, 18], although molecular markers have also been used. However, morphological identification is complicated, time-consuming and sometimes inaccurate, especially for specimens in nymphal stages, due to the wide variety of triatomines (over 140 species). Recently, with the rapid development of molecular biology techniques and bioinformatics, molecular identification based on specific DNA sequence analyses has become a common technological tool used to identify new species [19, 20], to provide rapid diagnoses of invasive pathogens [21] and cases of rare diseases [22], and to help in understanding the phylogenetic and phylogeographic relationships among species [23]. DNA markers including mitochondrial 16S and nuclear 28S ribosomal RNA (rRNA), mitochondrial cytochrome oxidase subunit I (COI), as well as DNA markers of nuclear origin, such as the internal transcribed spacer 2 (ITS-2) have been used to analyse genetic variation among triatomines, and explain their population structure and evolutionary history [6, 10, 23–28]. Therefore, this paper aims to use the above three universal markers for the identification of triatomine species found in Maoming, Guangdong Province and Zhangzhou, Fujian Province, China and to trace their potential origin through phylogenetic analysis.

## Materials and methods

### Insect collection and morphological identification

**Fig. 1:**
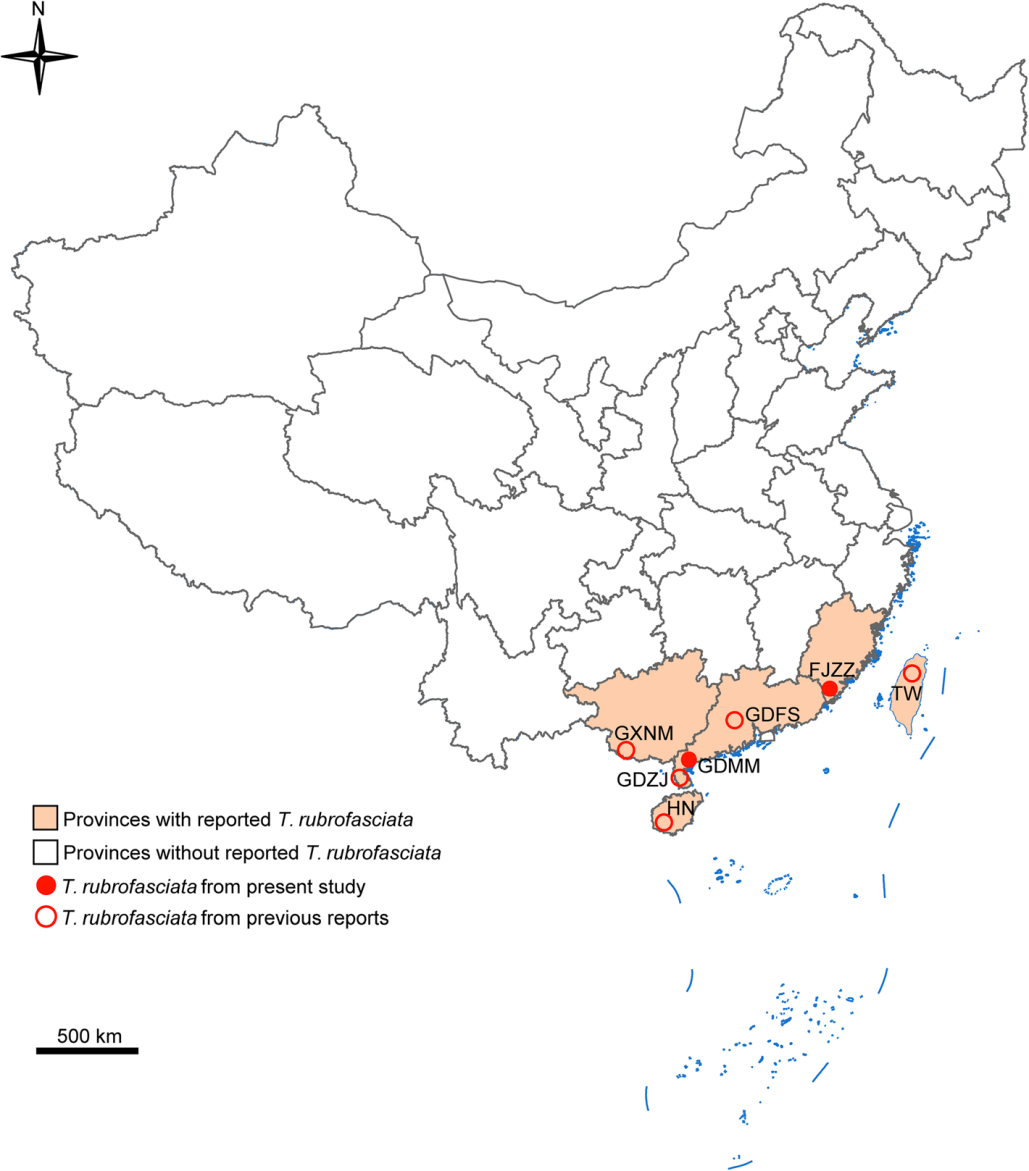
Map of distribution of *Triatoma rubrofasciata* caught in China in the present study or the previous reports. FJZZ: Zhangzhou City, Fujian Province; GDFS: Foshan City, Guangdong Province; GDMM: Maoming City, Guangdong Province; GDZJ: Zhanjiang City, Guangdong Province; GXNM: Ningming County, Guangxi Province; HN: Hainan Province; TW: Taiwan

Triatomine insects were actively searched for and caught using a butterfly net by technicians from Fujian Centre for Disease Control and Prevention, China. Captures took place in local residents’ houses close to each other in Huping Village (E 117°38′, N 24°39′), Fengshan Town, Hua’an County, Zhangzhou City, Fujian Province, China (FJZZ) in August 2017. Additionally, Dr. Min-Zhao Gao made captures in one dwelling (under wooden beds) in Tiantou Village (E 111°02′, N 21°33′), Shuidong Town, Dianbai District, Maoming City, Guangdong Province, China (GDMM) in August 2018 (Fig. 1). The locations chosen for collection were based on oral reports from local residents who found the insects in their houses. Triatomines were captured and transported live to a laboratory and identified using morphological features under a stereoscopic microscope (SZ650, Cnoptec, Chongqing, China) as described previously [29].

### DNA extraction, amplification, cloning and sequencing

Total genomic DNA was isolated from three legs of each triatomine specimen using the HiPure Tissue DNA Micro Kit (Cat No. D3125–02, Magen, Guangzhou, China) according to the manufacturer’s recommended protocol. The genes of mitochondrial 16S rRNA, COI and nuclear 28S rRNA were amplified using primers synthesized by Sangon Biotech (Shanghai, China), which are listed in Table 1.

**Table 1:** Primers used to amplify the mitochondrial 16S rRNA, COI genes and the nuclear ribosomal 28S rRNA gene

Gene target	Forward primer (5′ → 3′)	Reverse primer (5′ → 3′)	Expected length (bp)
16S rRNA	GGTTTTGAATGGCCGCAGTA	TCTCCGGTTTGAACTCAGATCA	491
28S rRNA	GCGAGTCGTGTTGCTTGATAGTGCAG	TTGGTCCGTGTTTCAAGACGGG	678
COI	TGTAGAAAGAGGAGCGGGAA	TCCGGTTTCCATGGCAATAA	439

Polymerase chain reactions (PCR) were performed in 50 μL of the following mixture: approximately 30 ng of DNA template, 1.1 × Golden Star T6 Super PCR Mix (Tsingke, China) and 0.4 μmol/L of each primer using a Bio-Rad PCR C1000 Touch instrument (Bio-Rad, USA). Negative controls were made with ultrapure water to control for contamination during PCR. For the 16S rRNA, 28S rRNA and COI genes, the fragments were amplified using the following thermal cycling conditions: initial denaturation at 98 °C for 2 min; 35 cycles of denaturation at 98 °C for 10 s, annealing at 55 °C for 30 s, extension at 72 °C for 15 s and final extension at 72 °C for 3 min. Afterwards, the PCR products were separated using 1% agarose gel electrophoresis, and the expected bands were excised under ultraviolet light, followed by purification with an Agarose Gel DNA Extraction Kit (Takara, Japan). The purified DNA fragments were inserted into a pClone007 Blunt Simple Vector (Tsingke, China), and the plasmids were used to transform *E. coli* DH5ɑ. Sequencing was performed using the vector primers (M13 forward and M13 reverse) in an Automated DNA Analyzer (ABI 3730XL, Applied Biosystems, Foster City, CA, USA) with the BigDye Terminator v3.1 Cycle Sequencing Kit (Cat No. 4337457, Applied Biosystems, Foster City, CA, USA) by Tsingke Biotechnology Ltd., Co. (Guangzhou, China).

### Sequence alignment and phylogenetic analysis

The sequences of the 16S rRNA, 28S rRNA and COI genes were submitted to the National Centre for Biotechnology Information (NCBI) GenBank database (https://www.ncbi.nlm.nih.gov/genbank/) under the accession numbers MK601647, MK601646, MK602653, MK602652, MK614012 and MK614011, respectively. They were submitted to GenBank for homology searches subsequent to triatomine species classification using BLAST (http://blast.ncbi.nlm.nih.gov/Blast.cgi) [30]. For the three genes, multiple alignments were performed among the best hit sequences using ClustalW2 [31] with default parameters; phylogenetic trees were constructed with MEGA 7.0 [32] using the Maximum Likelihood (ML) method [10] based on the Kimura 2-Parameter (K2P) nucleotide substitution model [33] and assessed with the bootstrap-resampling technique over 1000 replications. To demonstrate the polymorphism of *T. rubrofasciata* from multiple localities and their intraspecific genetic divergence, the pairwise genetic distances of 16S rRNA, 28S rRNA and COI were calculated between sequences obtained in this study and reference sequences downloaded from GenBank using the K2P nucleotide substitution model in MEGA 7.0 (www.megasoftware.net) [32].

## Results

### Morphological characteristics of *Triatoma rubrofasciata*

Four adult triatomine insects (two males and two females) were caught on a balcony where lumber was stored on the second floor of two farmers’ dwellings at Huping Village, Fengshan Town, Hua’an County (FJZZ). In addition, three adult triatomine insects (two males and one female) were captured under wooden beds in a living-room on the second floor of a local resident’s house at Tiantou Village, Shuidong Town, Dianbai District (GDMM).

**Fig. 2:**
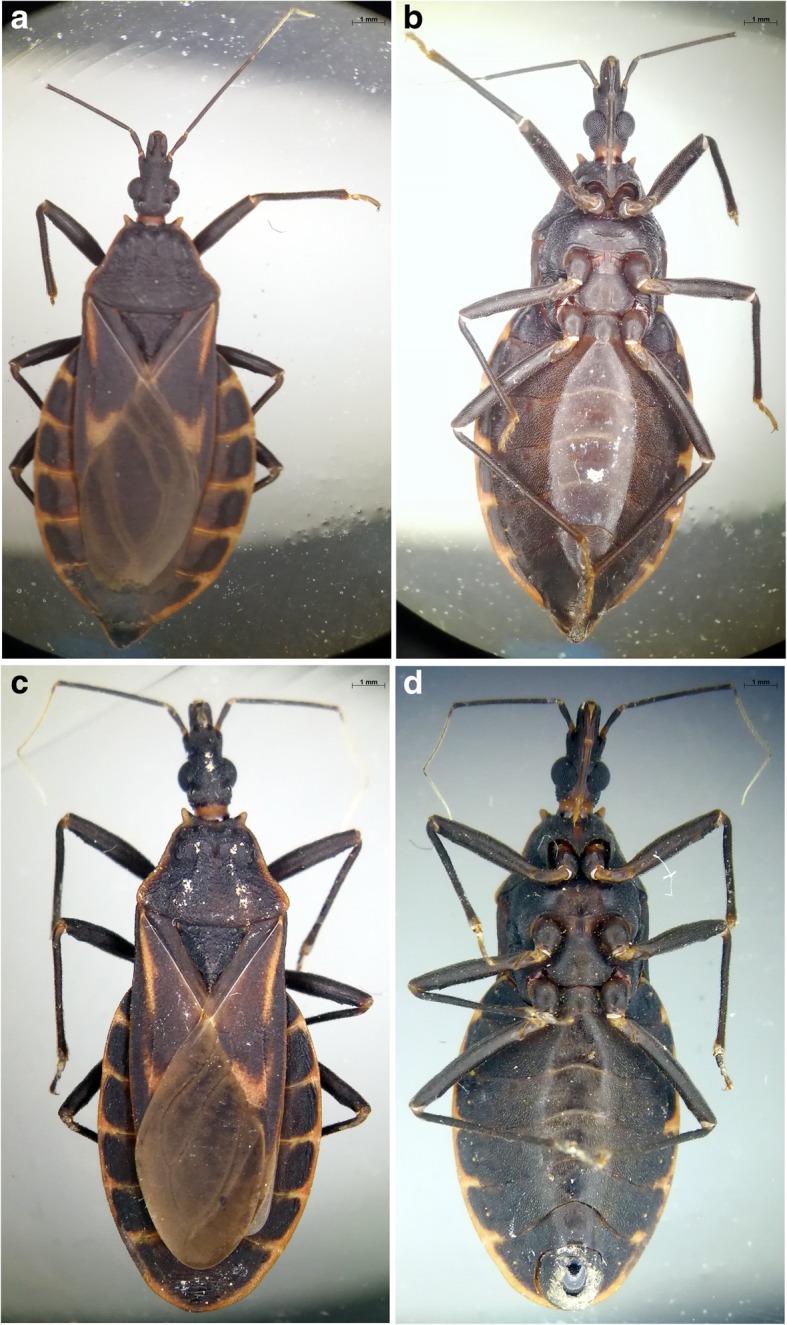
Dorsal and ventral views of the female (**a** and **b**) and male (**c** and **d**) adults of *Triatoma rubrofasciata*

**Fig. 3:**
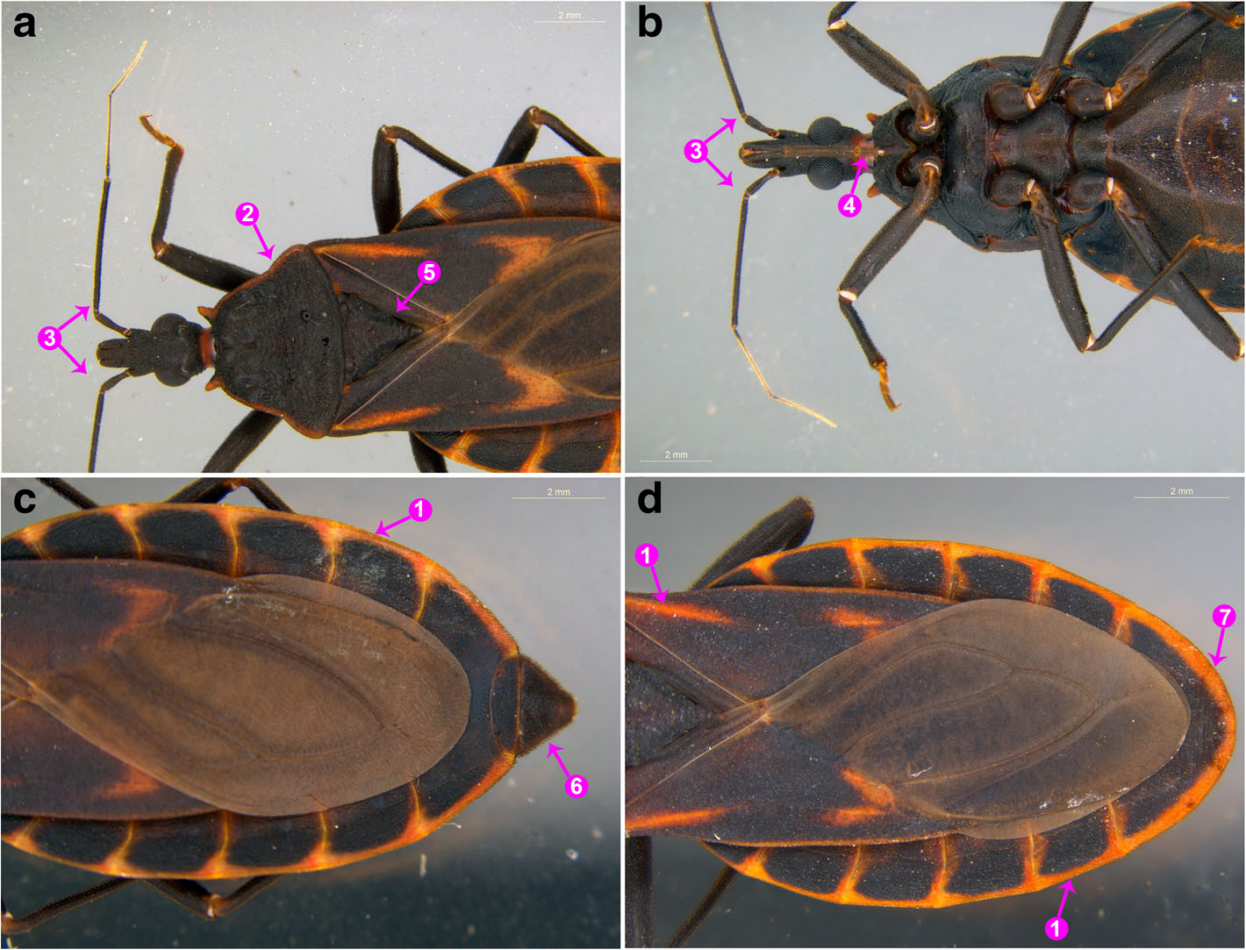
The key morphological characteristics of *Triatoma rubrofasciata*. **a** and **b**: Dorsal and ventral views of the head and thorax of *Triatoma rubrofasciata*; **c**: Dorsal view of the abdomen of the female *Triatoma rubrofasciata*; **d**: Dorsal view of the abdomen of the male *Triatoma rubrofasciata*. ①: Orange-red margin along the outer edge of abdomen and wings; ②: Orange-red margin along the side of pronotum; ③: The antennae were located between the compound eyes and clypeus while the 1st segment of antennae surpassed the apex of head; ④: Mouthpart with a long and straight proboscis covered with short hairs and the hairs became longer towards the tip; ⑤: The scutellum was broad and triangular to the tip; ⑥: The genital segment of female adult appeared in triangular shape; ⑦: The genital segment of male adult was in a broad circular shape

The triatomines collected in FJZZ and GDMM showed the morphological characteristics of *T. rubrofasciata* (Figs. 2 and 3). The specimens were black with orange-red margins along the lateral edge of the pronotum, wings and abdomen. The forepart of the pronotum was narrow, whereas the posterior was wide. The female and the adult males were approximately 23 mm and 21 mm in length respectively, with slightly elliptical shapes, and the long, cone-shaped heads were approximately three millimeter in length (Fig. 2). The thread-like antennae, located between the black compound eyes and clypeus, consisted of four segments, with the 1st segment surpassing the apex of head (Fig. 3a, b). Numerous small particles were densely distributed around the head and pronotum (Fig. 3a). The scutellum was triangular in shape with wrinkles in the middle and a sharp, needle-like apex (Fig. 3a). The genital segment of the adult female was triangular form, while that of the adult male had a broad circular shape (Fig. 3c, d).

### Molecular identification and phylogenetic relationships of *Triatoma rubrofasciata*

In this study, apart from morphological identification, we also used a molecular identification method based on sequences of 16S and 28S rRNA as well as COI to classify the triatomine species from GDMM and FJZZ. The results of the similarity analyses on the basis of the above genes demonstrated that the triatomine species in these two localities were accurately identified as *T. rubrofasciata* with 96–100% sequence matching identities.

For the 16S rRNA gene, *T. rubrofasciata* from FJZZ displayed the highest similarity with specimens from Foshan City, Guangdong Province, China (GDFS) with 99% identity. *T. rubrofasciata* from GDMM demonstrated the highest similarity with *T. rubrofasciata* from Zhanjiang City, Guangdong Province, China (GDZJ) with 99% identity. A polymorphism analysis of the 16S rRNA gene showed that two transition sites differed between the sequences of the insects from FJZZ and GDMM, and from FJZZ and GDZJ. Similarly, two distinctive transition sites were observed between the 16S rRNA sequences of the insects from GDMM and GDFS. Furthermore, one transversion site and one transition site differed between the 16S rRNA sequences of *T. rubrofasciata* from FJZZ and TW, while one transversion site and three transition sites differed between the sequences of the insects from GDMM and TW. However, only one distinguishing transition site was detected between the sequences of the insects from FJZZ and Vietnam and from GDMM and Vietnam. The genetic distance of the 16S rRNA gene ranged from 0.000 to 0.003 in *T. rubrofasciata* from the different locations, and the average intraspecific distance was 0.001 (Table 2, Additional file 2: Table S1).

**Table 2:** Pairwise intraspecific genetic distances of 16S rRNA gene of *Triatoma rubrofasciata*, based on the Kimura 2-Parameter model

	GDZJ	GDFS	GDMM	FJZZ	TW	Vietnam
GDZJ, China (MG674717)	–					
GDFS, China (KY420176)	0.000	–				
GDMM, China (MK601646)	0.000	0.000	–			
FJZZ, China (MK601647)	0.000	0.000	0.000	–		
TW, China (KP899112)	0.000	0.000	0.000	0.000	–	
Vietnam (HQ337019)	0.003	0.003	0.003	0.003	0.003	–

*GDZJ* Zhanjiang City, Guangdong Province, *GDFS* Foshan City, Guangdong Province, *GDMM* Maoming City, Guangdong Province, *FJZZ* Zhangzhou City, Fujian Province, *TW* Taiwan

For the 28S rRNA gene, both *T. rubrofasciata* from FJZZ and GDMM were similar, with *T. rubrofasciata* from GDZJ demonstrating 100% identity. The 28S rRNA sequences of *T. rubrofasciata* from FJZZ and GDMM were identical and only one distinctive transversion site was found between the sequences of *T. rubrofasciata* from FJZZ and GDFS, whereas no sites differed between the sequences of *T. rubrofasciata* from FJZZ and Ningming County, Guangxi Province, China (GXNM). In addition, two different transversion sites and one distinguishing transition site were found between the sequences of *T. rubrofasciata* from FJZZ and Brazil, while two transversion sites differed between the sequences of the triatomine insects from FJZZ and Vietnam. The genetic distance of the 28S rRNA gene in *T. rubrofasciata* from the different locations ranged from 0.000 to 0.002, and the average intraspecific distance was 0.001 (Table 3, Additional file 3: Table S2).

**Table 3:** Pairwise intraspecific genetic distances of 28S rRNA gene of *Triatoma rubrofasciata*, based on the Kimura 2-Parameter model

	GDZJ	GDFS	GDMM	FJZZ	GXNM	Vietnam	Brazil
GDZJ, China (MG675575)	–						
GDFS, China (KY420177)	0.000	–					
GDMM, China (MK602652)	0.000	0.000	–				
FJZZ, China (MK602653)	0.000	0.000	0.000	–			
GXNM, China (MH356281)	0.000	0.000	0.000	0.000	–		
Vietnam (KR632547)	0.000	0.000	0.000	0.000	0.000	–	
Brazil (KR632546)	0.002	0.002	0.002	0.002	0.002	0.002	–

*GDZJ* Zhanjiang City, Guangdong Province, *GDFS* Foshan City, Guangdong Province, *GDMM* Maoming City, Guangdong Province, *FJZZ* Zhangzhou City, Fujian Province, *GXNM* Ningming County, Guangxi Province

The COI gene of *T. rubrofasciata* from both FJZZ and GDMM presented the highest similarity with that from Brazil with 96% identity, with one variable site between them. Nevertheless, eighteen variable sites were detected between the COI sequences of the insects from FJZZ and Brazil, while nineteen variable sites were observed between the sequences of the insects from GDMM and Brazil. Thus, the genetic distance of the COI gene varied from 0.003 to 0.048, and the mean intraspecific distance was 0.032 (Table 4, Additional file 4: Table S3).

**Table 4:** Pairwise intraspecific genetic distances of COI gene of *Triatoma rubrofasciata*, based on the Kimura 2-Parameter model

	GDMM	FJZZ	Brazil
GDMM, China (MK614011)	–		
FJZZ, China (MK614012)	0.003	–	
Brazil (GQ869655)	0.048	0.045	–

*GDMM* Maoming City, Guangdong Province, *FJZZ* Zhangzhou City, Fujian Province

**Fig. 4:**
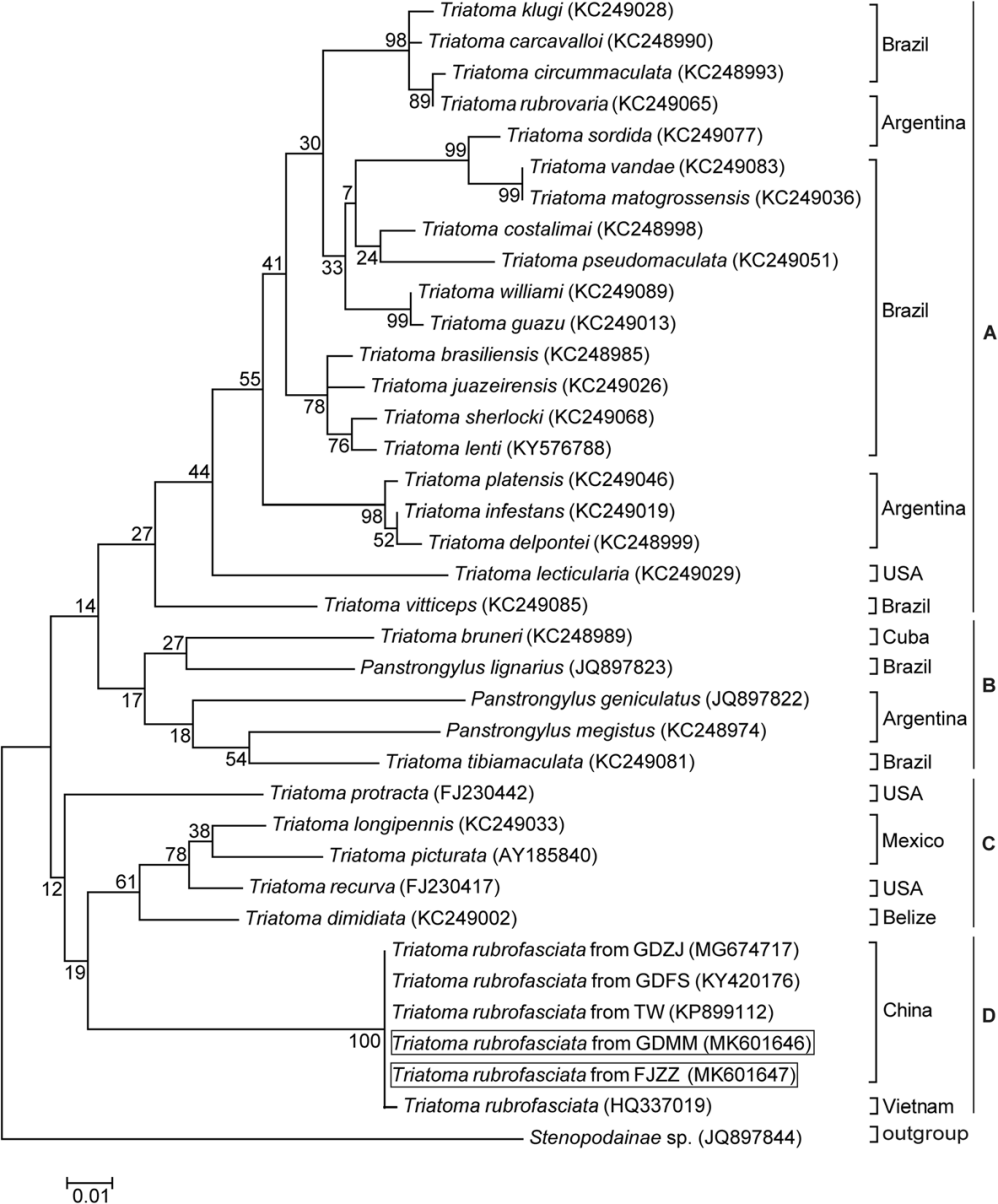
Phylogenetic tree of 16S rRNA gene among triatomine species. The tree was constructed by using the Maximum Likelihood method based on the Kimura 2-Parameter model. The relative values (%) on branches are based on 1000 bootstrap resamplings. *Stenopodainae* sp. was used to form the outgroup. *T. rubrofasciata* identified in the present study were shown in the square frame

**Fig. 5:**
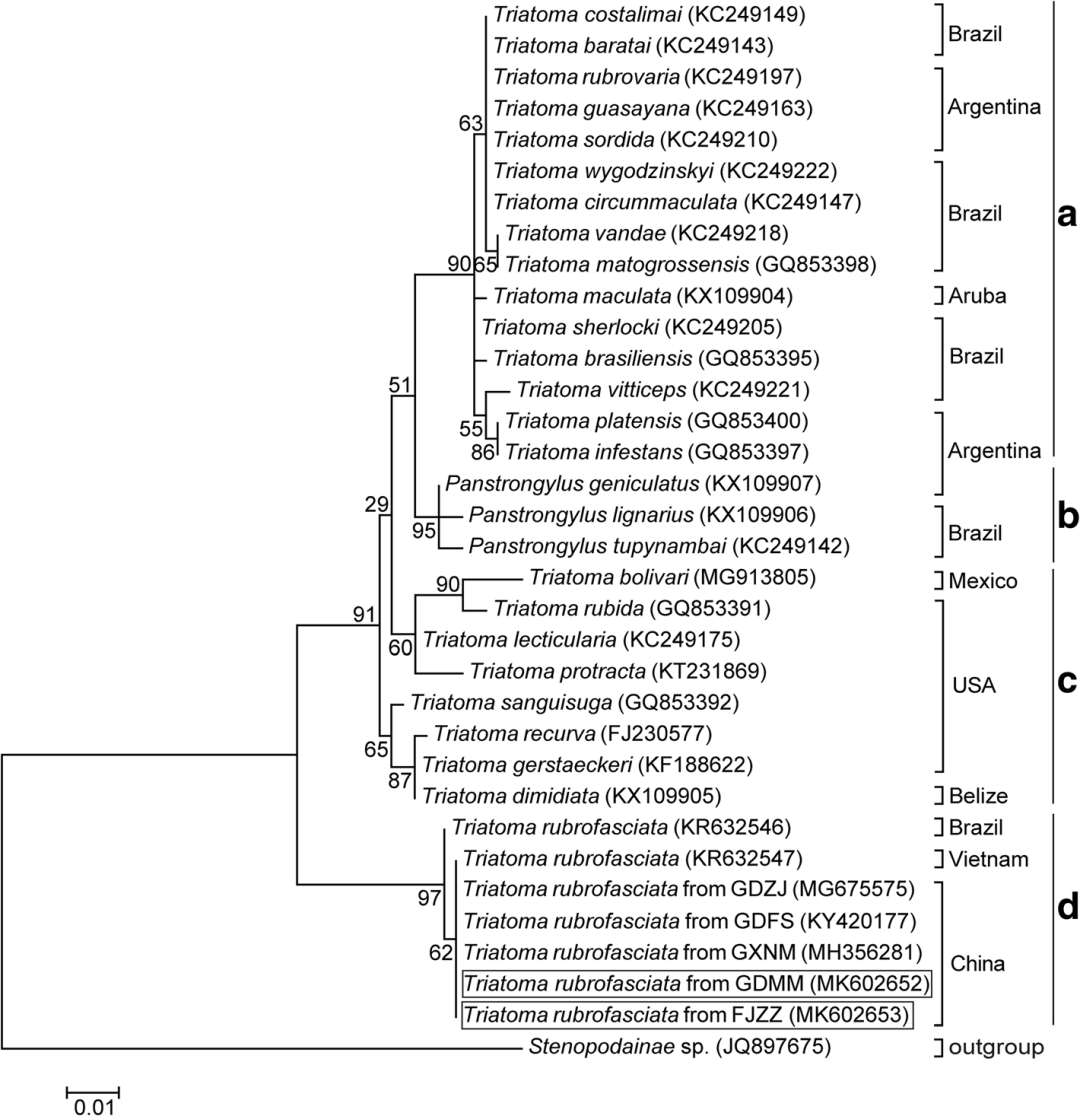
Phylogenetic tree of 28S rRNA gene among triatomine species. The tree was constructed by using the Maximum Likelihood method based on the Kimura 2-Parameter model. The relative values (%) on branches are based on 1000 bootstrap resamplings. *Stenopodainae* sp. was included in the trees as the outgroup. *T. rubrofasciata* identified in this study were shown in the square frame

**Fig. 6:**
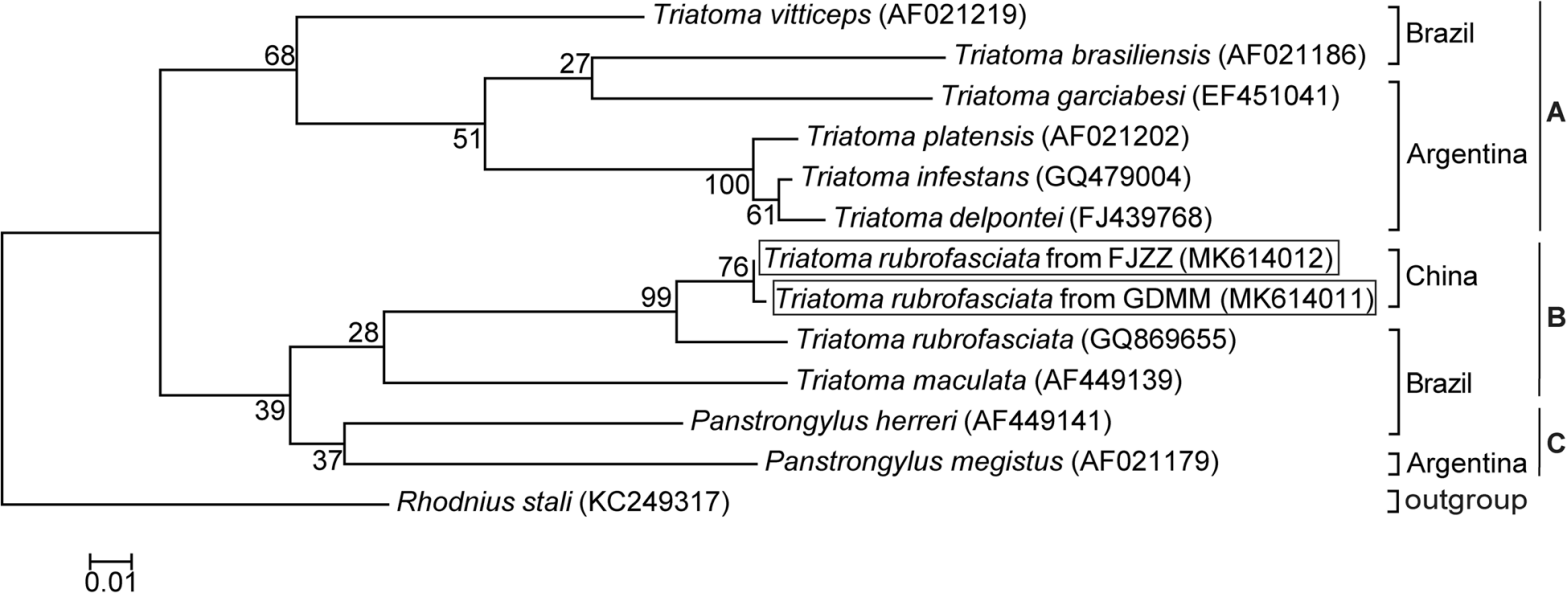
Phylogenetic tree of COI gene among triatomine species. The tree was constructed by using the Maximum Likelihood method based on the Kimura 2-Parameter model. The relative values (%) on branches are based on 1000 bootstrap resamplings. *Rhodnius stali* was set as the outgroup. *T. rubrofasciata* identified in this study were shown in the square frame

Furthermore, phylogenetic analysis of triatomine species based on the sequences obtained in this study and reference sequences with high similarity obtained from GenBank for the 16S rRNA, 28S rRNA and COI genes showed a distinct clade for all *T. rubrofasciata* individuals and was strongly supported by bootstrap values as high as 97 to 100%, which also confirmed that the insects captured in FJZZ and GDMM were *T. rubrofasciata* (Figs. 4, 5 and 6). Moreover, these results indicate that the triatomine species in the ML phylogenetic trees might be identified accurately using the barcode region used in this study.

According to the phylogenetic trees inferred from the 16S rRNA and 28S rRNA genes (Figs. 4 and 5), the Triatomini tribe is scattered throughout 4 main clades: (1) Clade A: South American *Triatoma,* including the *brasiliensis* subcomplex (*T. brasiliensis* from Brazil), the *rubrovaria* subcomplex (*T. rubrovaria* and *T. circummaculata* from Argentina), the *infestans* subcomplex (*T. infestans* from Argentina) and the *matogrossensis* subcomplex (*T. matogrossensis* and *T. costalimai* from Brazil); (2) Clade B: genus *Panstrongylus* (*P. geniculatus* and *P. megistus* from Argentina, and *P. lignarius* from Brazil); (3) Clade C: North and Central American *Triatoma*, including the *protracta* complex, (*T. protracta* from the United States of America), the *phyllosoma* subcomplex (*T. picturata* from Mexico) and the *dimidiata* subcomplex (*T. dimidiata* from Belize); and (4) Clade D: South American and Asian *Triatoma*, consisting of the *rubrofasciata* complex (*T. rubrofasciata* from China, Vietnam and Brazil). All of the Chinese triatomines collected from GDFS, GDZJ, GDMM, FJZZ and TW, along with the Vietnamese strain, were classified in the *rubrofasciata* complex and merged into Clade D, forming a distinctive clade with the *rubrofasciata* complex from Brazil, as shown in Fig. 5. This strongly suggests a close relationship between Asian triatomines and the *rubrofasciata* complex from Brazil. Similarly, phylogenetic analysis of Triatominae COI sequences demonstrated that Chinese triatomines from FJZZ and GDMM and the Brazilian *T. rubrofasciata* were in the same clade (Fig. 6). The Triatomini tribe was scattered throughout three clades of the South American *Triatoma* (genus *Panstrongylus*) as well as the South American and Asian *Triatoma*, due to the limited sequences of the COI gene available in GenBank (Fig. 6). These results strongly imply that Vietnam or Brazil is the most likely origin of the *T. rubrofasciata* in China.

## Discussion

*T. rubrofasciata* is a widespread haematophagous insect [34, 35], and it is the vector for *T. cruzi*, which causes Chagas disease and is a potential vector of bacteria and viruses [36]. It is likely distributed via shipping lines from the tropics and sub-tropics [34, 37, 38], and from inland regions in north-east India, Vietnam [35] and north-east Brazil [39]. Though *T. rubrofasciata* is the first species of the triatomine to have been described, its origins remain a mystery [40]. Several researchers have assumed an Old World (oriental) origin for *T. rubrofasciata* [29, 41]. However, more recent studies have suggested that all Triatominae are of a New World origin and that they were transported in association with rats (*Rattus norvegicus*) on ships sailing from the Americas [42–44].

Clear identification of invasive species is crucial for ecosystem maintenance, control, and risk assessment [19]. In this paper, the triatomine were identified as *T. rubrofasciata* by morphological analysis, which is supported by both the genetic and phylogenetic species concepts, indicating that 16S rRNA, 28S rRNA and COI can be used as specific gene markers for the reliable identification of triatomine species, as suggested by previous studies [6, 10, 44]. Morphological taxonomy and molecular identification are the two primary methods used for the identification of species [45–47]. Morphological similarities at both the intra- and inter-species level limit the usefulness of morphological taxonomic keys in some species [48]. Moreover, molecular identification might be effectively applied to classify the immature stages of triatomines, as traditional taxonomic methods are difficult to use when distinguishing nymphs, especially considering the species complexes that exhibit large morphological and chromatic variation [28]. However, molecular identification will not take the place of morphological systematics because it relies on species definitions produced from morphological analyses [49]; hence, both approaches should be applied to provide effective, efficient and accurate species identifications [50, 51], and the integration of morphological and molecular methods is indicated for species recognition of *T. rubrofasciata* in future entomological surveys in China [6].

To determine genetic variation in *T. rubrofasciata* from the different sites, genetic distances were calculated. The polymorphism analyses results showed that transitions were more frequent than transversions in the 16S rRNA, 28S rRNA and COI genes, and our data was consistent with observations from previous studies suggesting that COI is a faster evolving sequence, exhibiting higher variability than 16S or 28S rRNA [52, 53]. Hence, we suggest that 16S rRNA, 28S rRNA as well as COI genes are suitable for species identification while for tracking back the origin of the species, COI gene is applicable due to the higher genetic diversity.

Despite a relevant body of research on taxonomy, distribution, pathology and prevention [13], little is known about the phylogeny of *T. rubrofasciata* in Asia, as DNA sequence information regarding this vector is scarce. While an increasing number of *T. rubrofasciata* have occurred in southern China [6, 9–12], their origins remain unclear. Maximum Likelihood phylogenetic trees inferred by 16S rRNA and 28S rRNA gene sequences suggest that the *Triatoma* could be divided into four clades, not three as suggested by a previous study that used a limited sample size [9], and that the *T. rubrofasciata* from China, Vietnam and Brazil form a new, cohesive clade, indicating a close relationship between *T. rubrofasciata* from Asia and South America, with no incipient speciation between them.

Though the interrelationships among genera and species of the Triatomini have been shown as unstable in previous studies [43, 44], most researchers consider the Asian species of *T. rubrofasciata* to be derived from the Americas and form a monophyletic clade within the Triatominae [4, 44], which is consistent with the findings from this study. The results of the comparative analyses of the 16S rRNA, 28rRNA and COI sequences of *T. rubrofasciata* exhibited high genetic similarity, suggesting a very recent dispersal of this invasive insect in Asia (including China and Vietnam) and a derivation of the insect from South America, primarily from Brazil.

Though this study does allow a better understanding of the phylogenetic relationships among triatomine species and determine the phylogenetic origin approximation of *T. rubrofasciata* in China, extensive population genetic study is needed to confirm these results due to the small sample size and limited sampling area in this paper. Especially for the COI gene, we can only obtain the sequences of *T. rubrofasciata* from FJZZ and GDMM in China as well as Brazil, but not Vietnam, from NCBI GenBank database. Moreover, the morphological difference between the bugs from China, Vietnam or Brazil is missed in the present study because the authors have not the samples of *T. rubrofasciata* captured in Vietnam and Brazil.

## Conclusions

This is the first report of the triatomine species found in GDMM and FJZZ in China, which likely originated in Vietnam or Brazil. The current molecular taxonomic study and phylogenetic origin analysis revealed genetic data of *T. rubrofasciata* from different localities in China, which could contribute to the establishment of adequate vector control strategies and adding phylogenetic information regarding this widespread, invasive insect of medical significance.

## Additional files

Additional file 1: Multilingual abstracts in the five official working languages of the United Nations. (PDF 597 kb)

Additional file 2: **Table S1.** Genetic distance table for 16S rRNA sequences used in Fig. 4. Standard error estimates were shown above the diagonal. Analyses were conducted using the Kimura 2-Parameter model. (XLSX 26 kb)

Additional file 3: **Table S2.** Genetic distance table for 28S rRNA sequences used in Fig. 5. Standard error estimates were shown above the diagonal. Analyses were conducted using the Kimura 2-Parameter model. (XLSX 20 kb)

Additional file 4: **Table S3**. Genetic distance table for COI sequences used in Fig. 6. Standard error estimates were shown above the diagonal. Analyses were conducted using the Kimura 2-Parameter model. (XLSX 11 kb)

## Data Availability

Gene data in the present study are being submitted to the National Center for Biotechnology Information (NCBI).

## Abbreviations

COI: cytochrome oxidase subunit I
FJZZ: Zhangzhou City, Fujian Province, China
GDFS: Foshan City, Guangdong Province, China
GDMM: Maoming City, Guangdong Province, China
GDZJ: Zhanjiang City, Guangdong Province, China
GXNM: Ningming County, Guangxi Province, China
ITS-2: internal transcribed spacer 2
LAC: Latin American and Caribbean
ML: Maximum Likelihood
NCBI: National Center for Biotechnology Information
PCR: polymerase chain reaction
rRNA: ribosomal RNA
TW: Taiwan
