# Supplementary material for: Taxonomic integrative and phylogenetic identification of the first recorded Triatoma rubrofasciata in Zhangzhou, Fujian Province and Maoming, Guangdong Province, China
Source: Infect Dis Poverty. 2019 Aug 13;8:70. doi: 10.1186/s40249-019-0579-8 (PMC6693202; doi:10.1186/s40249-019-0579-8)
Supplement: Supplementary file 1 — Multilingual abstracts in the five official working languages of the United Nations. (PDF 597 kb) [file 40249_2019_579_MOESM1_ESM.pdf]

Translation of the abstract into the five official working languages of the United Nations

التعيين السلالي والتصنيفي التكاملي لأول *ترياتوما ريبروفاشياتا* مُسجلة في زهانغزهو، محافظة فوجيان، ماومينغ، محافظة غوانغدونغ، الصين

يو هو، مين تشاو قاو، بينغ هوانغ، هونغ لي تشو، يو بن ما، مين يو زهو، شاو يون تشينغ، هان قوه شيه، زهي يو لف

نبذة مختصرة

خلفية: تعيش أغلب أنواع الترياتوما بالتحديد في أمريكا اللاتينية. بالرغم من ذلك، ثمة نوع واحد، ترياتوما ريبروفاشياتا, *تم تسجيله بالأمريكتين وكذلك في مناطق ساحلية شتى بأفريقيا وآسيا. في*السنوات الأخيرة تم الإعلان عن تزايد أعداد ت. ريبروفاشياتا في جنوب الصين. على الرغم من ذلك، ما زال مصدر الحشرات الناقلة الغازية تلك مجهولاً في الصين. ولذلك، ثمة حاجة ماسة إلى التعيين الدقيق والتحليل التصنيفي للحشرات.

الطرق والأساليب: إجمالي سبعة ترياتومينتم العثور على عينات الحشرات وجمعها من مدينة ماومينغ، محافظة غوانغدونغ، الصين (GDMM) ومدينة زهانغزهو، محافظة فوجيان، الصين (FJZZ)، تباعاً رُصدت عينات الحشرات الناقلة التي تم الحصول عليها تحت المجهر التشريحي من أجل إجراء التصنيف الشكلي وبعد ذلك تم استخراج الحمض النووي الجينومي، الحمض النووي الريبوزي الريبوسومي 16س (rRNA)، الحمض النووي الريبوزي الريبوسومي 28س، كما تم سلسلة وتكبير الجين الخاص بالعينات والذي يحمل اسم وحيدة أوكسيداز السيتوكروم رقم 1. فيما بعد، تم إجراء التحاليل السلالية الجزيئية بناء على التسلسلات المتعددة للجينات المذكورة أعلاه من أجل التعرف على الأنواع وتعيين مقاربة الأصل السلالي بشكل دقيق.

النتائج: حشرات الترياتومين التي تم الحصول عليها من GDMM و FJZZ تم التعرف عليها مثل ترياتوما ريبروفاشياتا *باستخدام* التحاليل الجينية والشكلية. جميع حشرات ت. ريبروفاشياتا الصينية التي تم الحصول عليها من FJZZ، GDMM، والأماكن الأخرى بجنوب الصين، جنباً إلى جنب السلالات البرازيلية والفيتنامية شكلت *فرع حيوي مترابط. من المحتمل أنت.ريبروفاشياتا* المتواجدة في GDMM و FJZZ متفرعة من السلالات التي عُثر عليها في البرازيل أو فيتنام.

الاستنتاجات: وفقاً لأفضل ما نعرفه، فإن هذا هو التسجيل الأول من نوعه للحشرة الغازية *ت. ريبروفاشياتا*، والتي من المحتمل أنها تتفرع من سلالات أصلية للبرازيل أو فيتنام، في كل من مدينة ماومينغ، محافظة غوانغدونغ، مدينة زهانغزهو، محافظة فوجيان بالصين. مقارنة تسلسلات الحمض النووي لكل من الحمض النووي الريبوزي الريبوسومي 16س، الحمض النووي الريبوزي الريبوسومي 28س، وجينات وحيدة أوكسيداز السيتوكروم رقم 1 أكدت التعيين النوعي ل *ت. ريبروفاشياتا*،ومنشأها المحتمل في الصين بناء على التحاليل السلالية التي أجريت في هذه الدراسة. ثمة حاجة ملحة لمزيد من التدخلات الموجهة والرصد الحشري المحسن من أجل السيطرة على انتشار هذه الحشرة*الآكلة للدم* في الصين.

Translated from English version into Arabic by Mohamed Bonna, proofread by Amal Alaboud, through

TRANSLATORS
WITHOUT BORDERS

中国福建省漳州市与广东省茂名市首次发现的红带锥蝽的综合性分类鉴定与系统发生学研究

胡玥，高敏照，黄萍，周洪利，马玉斌，周旻昱，成韶芸，谢汉国，吕志跃

摘要

引言：锥蝽可传播美洲锥虫病，且大多数种类仅分布于拉丁美洲。唯有红带锥蝽广泛分布于美洲以及非洲、亚洲的部分地区。近年来，华南多个地区陆续发现这种外来入侵物种，然而其来源仍不清楚，因此亟需对入侵中国的红带锥蝽进行准确鉴定并进行系统发生学研究，便于对其进行科学有效的防制。

方法：分别将从中国广东省茂名市及福建省漳州市捕获的 7 只锥蝽首先在解剖镜下进行形态学鉴定，再提取这些昆虫的 DNA 作为模板，以 16S 核糖体 RNA（16S rRNA）、28S rRNA 及细胞色素氧化酶亚基I（COI）基因特异性引物进行扩增、测序并与亲缘关系相近的锥蝽序列进行多序列比对，进行分子鉴定及溯源分析。
结果：形态学鉴定结合分子鉴定技术均证实广东省茂名市和福建省漳州市采集到的锥蝽都属于红带锥蝽。将此两处红带锥蝽与来自亚洲其他地区以及美洲的红带锥蝽进行种系发生学研究，结果显示，亚洲（包括中国境内目前报道的）红带锥蝽与美洲的红带锥蝽可单独形成一个新的分化枝，且中国的红带锥蝽可能源自于巴西和（或）越南。
结论：本文首次报道在广东茂名与福建漳州发现入侵生物——红带锥蝽。基于 16s rRNA、28s rRNA 及 COI 基因序列的分析不仅可结合形态学观察对红带锥蝽进行准确的物种鉴定，还可进行系统发生学研究，推测其可能来源。针对性的干预及加强监控将有助于控制红带锥蝽在中国的进一步扩散。

Translated from English version into Chinese by Yue Hu, Zhi-Yue Lv

Identification phylogénétique et intégrative taxonomique des premières *Triatoma rubrofasciata* répertoriées à Zhangzhou, province du Fujian et à Maoming, province de Guangdong, Chine

Yue Hu, Min-Zhao Gao, Ping Huang, Hong-Li Zhou, Yu-Bin Ma, Min-Yu Zhou, Shao-Yun Cheng, Han-Guo Xie, Zhi-Yue Lv

Résumé

Contexte: La plupart des espèces de Triatominae vivent exclusivement en Amérique latine. Toutefois, une espèce, la *Triatoma rubrofasciata*, a été répertoriée dans les Amériques ainsi que dans diverses régions portuaires d’Afrique et d’Asie. Ces dernières années, dans le sud de la Chine, un nombre croissant de *T. rubrofasciata* a été rapporté. Cependant, l’origine de la présence de cet insecte vecteur invasif en Chine reste à déterminer. Par conséquent, il est urgent de procéder à une identification précise et aux analyses phylogénétiques de ces insectes.
Méthodes: En tout, nous avons trouvé et recueilli sept spécimens de triatome à Maoming, dans la province de Guangdong (GDMM) et à Zhangzhou, dans la province de Fujian (FJZZ) en Chine. Nous avons observé les spécimens d’insecte vecteur recueillis sous un microscope de dissection afin de les classer en fonction de leur morphologie et nous avons extrait leur ADN génomique. Ensuite, nous avons procédé à l’amplification et au séquençage des ARN ribosomaux (ARNr) 16S et 28S et des gènes de la sous-unité I de la cytochrome-oxydase (COI) de ces espèces. Par la suite, nous avons procédé à des analyses phylogénétiques moléculaires basées sur les alignements multiples des gènes mentionnés ci-dessus afin d’identifier les espèces et de déterminer de façon précise, l’approximation de l’origine phylogénétique.
Résultats: Grâce aux analyses génétiques et morphologiques, les triatomes prélevés à GDMM et FJZZ ont été identifiés comme appartenant à l’espèce *Triatoma rubrofasciata*. Tous les insectes *T. rubrosfasciata* de souche chinoise recueillis à FJZZ, GDMM et dans d’autres villes du sud de la Chine, ainsi que les souches vietnamiennes et brésiliennes, forment un nouveau clade homogène. L’espèce *T. rubrofasciata* provenant de GDMM et de FJZZ semble être dérivée de souches vietnamiennes et brésiliennes.
Conclusions: À notre connaissance, il s’agit là du premier signalement de l’insecte invasif *T.*

rubrofasciata à Maoming et à Zhangzhou, dans les provinces de Guangdong et de Fujian, en Chine. Il semblerait que cet insecte soit dérivé d'une souche provenant du Vietnam ou du Brésil. Une comparaison des séquences ADN des gènes ARNr 16S, ARNr 28s et COI a confirmé l'identification spécifique de l'espèce *T. rubrofasciata*. L'hypothèse de son origine chinoise est fondée sur les analyses phylogénétiques réalisées dans le cadre de cette étude. Il est urgent de procéder à des interventions plus ciblées et d'améliorer la surveillance entomologique afin de maîtriser la propagation de cet insecte hématophage en Chine.

Translated from English version into French by Florie Ulian, proofread by Michael Seiagam, through

TRANSLATORS
WITHOUT BORDERS

Таксономическая, интегративная и филогенетическая идентификация первых зарегистрированных клопов вида *Triatoma rubrofasciata* в г. Чжанчжоу, провинции Фуцзянь, в Китае и в г. Маомин, провинции Гуандун, в Китае

Yue Hu, Min-Zhao Gao, Ping Huang, Hong-Li Zhou, Yu-Bin Ma, Min-Yu Zhou, Shao-Yun Cheng, Han-Guo Xie, Zhi-Yue Lv

Аннотация

Справочная информация: Большинство видов триатомовых клопов распространено исключительно в Латинской Америке. Однако сообщалось о появлении одного вида*Triatoma rubrofasciata*,в Северной и Южной Америке, а также в различных портовых зонах в Африке и Азии. В последние годы поступали сообщения о значительномувеличении числа клопов вида *T. rubrofasciata* в южных районах Китая. Несмотря на это причина возникновения данного инвазивного вектора насекомых в Китае остается неизвестной, поэтому необходимо срочно провести точную идентификацию и филогенетический анализ этих клопов.

Методы: Всего было найдено и собрано семь особей вида триатомовых клопов в г. Маомин, провинции Гуандун, в Китае (GDMM) и г. Чжанчжоу, провинции Фуцзянь, Китае (FJZZ) соответственно. Полученные образцы насекомых-векторов наблюдались под рассекающим микроскопом для морфологической классификации, а затем была выделена геномная ДНК, и гены 16S рибосомальной РНК (рРНК), 28S рРНК, а также субъединицы I цитохромоксидазы (COI) этого вида были амплифицированы и секвенированы. Впоследствии были проведены молекулярные филогенетические анализы на основе множественного выравнивания вышеупомянутых генов с целью достоверной идентификации видов и определения филогенетического происхождения.

Результаты: Триатомовые насекомые, собранные в GDMM и FJZZ, были идентифицированы как клопы вида *Triatoma rubrofasciata* с помощью морфологического и генетического анализов. Все китайские клопы вида *T. rubrofasciata*, собранные в FJZZ, GDMM и другой местности в южной части Китая, вместе с вьетнамским и бразильским штаммом, образуют новый, целостный класс. Вероятно, вид *T. rubrofasciata* в GDMM и FJZZ произошел от штаммов, находящихся во Вьетнаме или Бразилии.

Выводы: Насколько нам известно, это первое сообщение о появлении инвазивных насекомых вида *T. rubrofasciata*, которые, вероятно, произошли от штаммов, зародившихся во Въетнаме или Бразилии, как в г. Маомин, провинции Гуандун, так и в г. Чжанчжоу, провинции Фуцзянь в Китае. Сравнение последовательностей ДНК генов 16s рРНК, 28s рРНК и COI подтвердило специфическую идентификацию вида*T. rubrofasciata*, , а его потенциальное происхождение в Китае основано на филогенетических анализах, проведенных в этом исследовании. Для контроля за распространением этих насекомых, питающихся кровью (гематофагов) в Китаесрочно необходимы более целенаправленные вмешательства и усовершенствованный энтомологический надзор.

Translated from English version into Russian by Veronika Demeshchyk, proofread by Anna Kukharchuk, through

Identificación taxonómica integrativa y filogenética del primer registro *Triatoma rubrofasciata* en Zhangzhou, Provincia de Fujian y Maoming, Provincia de Guangdong, China

Yue Hu, Min-Zhao Gao, Ping Huang, Hong-Li Zhou, Yu-Bin Ma, Min-Yu Zhou, Shao-Yun Cheng, Han-Guo Xie, Zhi-Yue Lv

Resumen

Contexto: La mayoría de las especies Triatominae viven, exclusivamente, en Latinoamérica. Sin embargo, se ha registrado una especie en las Américas, *Triatoma rubrofasciata*, así como en varias zonas portuarias de África y Asia. Se ha registrado un creciente número de *T. rubrofasciata* , en el sur de China y durante los últimos años. Sin embargo, el origen de este insecto vector invasivo, en China, sigue siendo desconocido; por lo tanto, son, urgentemente, necesarios una identificación precisa y un análisis filogenético de los insectos.

Métodos: Se encontraron y recolectaron un total de siete especímenes de triatominos, en la ciudad de Maoming, provincia de Guangdong, China (GDMM) y en la ciudad de Zhangzhou, provincia de Fujian, China (FJZZ), respectivamente. Las muestras de vectores de insectos obtenidas se observaron de acuerdo con un microscopio de disección, para la clasificación morfológica. A continuación, se extrajo el ADN genómico y se amplificaron y secuenciaron los genes del ARN ribosomal 16S (ARNr), ARNr 28S y la subunidad I (COI) del citocromo oxidasa de la especie. Posteriormente, se realizaron análisis filogenéticos moleculares, basados en múltiples alineaciones de los genes anteriores, para identificar las especies y determinar la aproximación del origen filogenético con precisión.

Resultados: Los insectos triatominos recolectados de GDMM y FJZZ se identificaron como *Triatoma rubrofasciata* utilizando análisis morfológicos y genéticos. Todos los *T. rubrofasciata* chinos capturados en FJZZ, GDMM y otras localidades del sur de China, junto con una cepa vietnamita y brasileña, formaron un nuevo clado cohesivo. *T. rubrofasciata* en GDMM y FJZZ, probablemente, se deriven de cepas que se encontraron, en Vietnam o en Brasil.

Conclusiones: Según nuestro conocimiento, este es el primer registro del insecto invasor *T.*

rubrofasciata(probablemente derivado de cepas nativas de Vietnam o Brasil), tanto en la ciudad de Maoming, provincia de Guangdong, como en la ciudad de Zhangzhou, provincia china de Fujian. Una comparación de las secuencias de ADN de los genes rRNA 16s, rRNA 28s y COI confirmó la identificación específica de *T. rubrofasciata*, y su posible origen, en China, se basa en los análisis filogenéticos que se realizaron en este estudio. Se necesitan, urgentemente, intervenciones más específicas y una mejor vigilancia entomológica, para controlar la propagación de este insecto hematófago, en China.

Translated from English version into Spanish by Inmaculada Espárrago, proofread by María Luz Puerta, through

TRANSLATORS
WITHOUT BORDERS
